# Practical recommendations for measuring rates of visual field change in glaucoma

**DOI:** 10.1136/bjo.2007.135012

**Published:** 2008-01-22

**Authors:** B C Chauhan, D F Garway-Heath, F J Goñi, L Rossetti, B Bengtsson, A C Viswanathan, A Heijl

**Affiliations:** 1 Department of Ophthalmology and Visual Sciences, Dalhousie University, Halifax, Canada; 2 Moorfields Eye Hospital and UCL and Institute of Ophthalmology NIHR Biomedical Research Centre, London, UK; 3 Barcelona Glaucoma Centre, USP-Instituto Oftalmológico de Barcelona, Barcelona, Spain; 4 Department of Ophthalmology, Eye Clinic, University of Milan, San Paolo Hospital, Milan, Italy; 5 Department of Ophthalmology, Malmo University Hospital, Lund University, Lund, Sweden

## Abstract

To date, there has been a lack of evidence-based guidance on the frequency of visual field examinations required to identify clinically meaningful rates of change in glaucoma. The objective of this perspective is to provide practical recommendations for this purpose. The primary emphasis is on the period of time and number of examinations required to measure various rates of change in mean deviation (MD) with adequate statistical power. Empirical data were used to obtain variability estimates of MD while statistical modelling techniques derived the required time periods to detect change with various degrees of visual field variability. We provide the frequency of examinations per year required to detect different amounts of change in 2, 3 and 5 years. For instance, three examinations per year are required to identify an overall change in MD of 4 dB over 2 years in a patient with average visual field variability. Recommendations on other issues such as examination type, strategy and quality are also made.

## STATEMENT OF INTEREST

Assessment of visual field damage is the major index of the functional impact of glaucoma with direct relevance to quality of life measures.^1^^–^6 Visual field change was used as a primary endpoint for progression in the recent glaucoma trials,^7^^–^12 and its measurement is the cornerstone of glaucoma management influencing therapeutic decisions.

The objective of this perspective is to provide practical recommendations for measuring clinically relevant rates of glaucomatous visual field progression to help identify patients at risk for visual impairment. They focus on the frequency of examinations required for detecting various amounts and rates of visual field change.

## STATEMENT OF NEED

In routine clinical glaucoma practice, the frequency of visual field examinations varies significantly^13^ and usually falls substantially below recommendations for maintaining minimum practice standards.^14^ An unacceptable number of glaucoma patients become visually impaired or even blind while under care.^15^^–^21 Several factors including over-reliance on intraocular pressure (IOP) to measure treatment adequacy could contribute to this finding. Having sufficient visual field data and analysis methods may help the ophthalmologist deliver more targeted care resulting in better functional outcomes.

There are three main barriers to performing sufficiently frequent visual field examinations.

### Data volume and analysis

Standard automated perimetry (SAP) generates large volumes of data, which may be time-consuming and difficult to interpret without statistical software. There are no generally recommended methods for usage of the analyses and translating them to clinical practice.

### Resource allocation

Financial resources allocated to perimetry are often inadequate and vary across jurisdictions. The need for more frequent and regular visual field examinations has been poorly emphasised, perhaps because of the lack of a strong evidence-based rationale to monitor disease progression.

### Definition of progression

While criteria for progression have been clearly defined in clinical trials, the identical criteria may not be applicable to clinical practice. The lack of a definition of clinically significant progression and how to use it practically may be a disincentive to performing frequent examinations. In turn, clinical decisions could be made with inadequate information, or serious progression could be missed because of an insufficient number of examinations.

## METHOD TO MEASURE VISUAL FIELD PROGRESSION

### Standard automated perimetry (SAP) should be used

Knowledge on visual field progression in glaucoma has come almost exclusively from SAP. Recent clinical trials in glaucoma have used only SAP as a primary functional endpoint, and clinical benefit from their findings is easiest if SAP is used.

### What to measure

There are several approaches to analysing visual field progression, including event (comparison of a follow-up examination to a baseline) and trend (regression analyses to measure rates of change) based methods, each with a myriad of variations. Analyses can be subdivided into individual points, visual field sectors, or the whole field. For the purpose of simplicity and universality, it is recommended that rates of change are measured in units of mean deviation or mean defect (MD), the average pointwise difference between a given test result and the normal age matched reference value.

### Newer forms of testing should not be performed at the expense of SAP

It is recognised that patients can only perform a limited number of tests during clinic visits, and the number of patient visits is limited by physician patient loads, reimbursement and other resource considerations. While tests such as short-wavelength automated perimetry (SWAP) and frequency-doubling technology (FDT or Matrix) perimetry can provide additional or confirmatory evidence, they remain in the clinical research domain. In routine clinical practice, they should not be conducted at the expense of SAP examinations. Similarly, new imaging techniques of the optic disc and retinal nerve fibre (RNFL) are widely used in some clinical practices and provide complementary information. However, they cannot substitute perimetry as a surrogate measure of visual field change.

## NUMBER AND FREQUENCY OF VISUAL FIELD EXAMINATIONS

Visual field examinations should be conducted in all glaucoma patients and suspects. The appropriate frequency and intervals set out below are determined by the rate of change to be detected and measurement variability. These recommendations specify the *minimum* requirements. There are many circumstances when the frequency of examinations should be increased because of a higher perceived risk of functional loss—for example, suspicion of optic disc change, inadequate IOP control, advanced field damage, pseudoexfoliation, increased age and morbidity in the fellow eye.

It is important to obtain sufficient number of reliable examinations in the early follow-up to establish a good baseline and rule out rapid progression. Clinicians should take advantage of the more frequent appointments in the first year and, if possible, perform a visual field examination at each visit.

Rates of MD change in most glaucoma patients vary from 0 to −2.5 dB/year, depending on the severity of disease, treatment and population samples.^22^^–^25 They are pragmatic estimates of visual disability patients can suffer at given rates of progression and baseline damage. For example, a patient with early loss (MD = −4 dB) and a rapid rate of progression (−2 dB/year) can be expected to have total visual disability (−30 dB) in 13 years. In a patient with moderate loss (−12 dB), that rate of change will have the same outcome in 9 years, while slower progression (−0.2 dB/year) is unlikely to lead to blindness in that patient’s lifetime.

**Figure 1:**
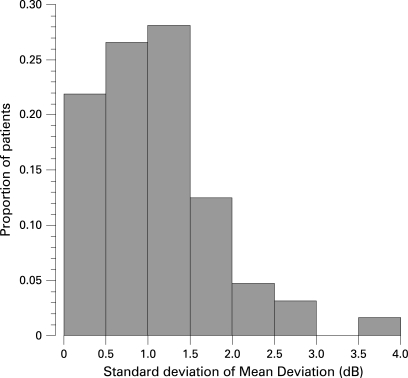
Distribution of intra-individual standard deviation (SD) of MD values in patients followed for up to 12 years.

The ability (statistical power) to detect a given rate of visual field change expressed in MD/year will depend on the variability of MD over time, the number of examinations and the amount of change we wish to detect. Using the distribution of standard deviations (SD) of MD from glaucoma patients in a longitudinal study^26^ (fig 1), low, moderate and high variability are defined as an SD of 0.5, 1 and 2 dB, respectively.

**Figure 2:**
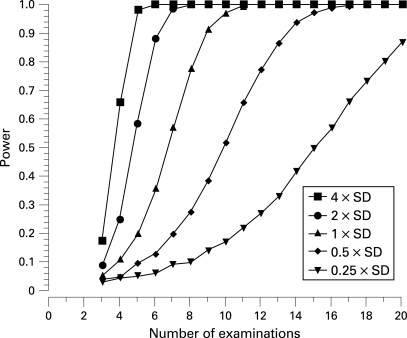
Statistical power to detect a significant rate of change of MD expressed as a function of variability (SD of the MD values) and number of examinations.

The statistical power to detect various rates of MD change (expressed as a multiple of the SD) for given numbers of examinations is shown in fig 2. Detecting a very rapid rate (four times the SD of MD variability) of change with 80% power requires between four and five examinations. In other words, such a magnitude of change will be detected four times out of five with between four and five examinations. On the other hand detecting a small rate of change (1/4 of the SD of MD variability) requires 19 examinations for the same power.

In practical terms, these data can be expressed as the time period required to detect MD deterioration of −0.5 dB/year, −1.0 dB/year and −2.0 dB/year with 80% power (table 1) with one, two and three examinations per year. These data clearly show that detecting rates of visual field change with certainty generally requires many visual fields; however, larger rates of change require fewer examinations. In a patient with rapid progression (−2 dB/year) and low variability, the time required to detect change with 80% power is 5 years if examinations are performed once a year, 2.5 years with two examinations per year and as little as 1.7 years if examinations are performed three times per year. In a more typical scenario with moderate field progression (−0.5 dB/year) and moderate variability, the respective times to detection are 13, 6.5 and 4.3 years for one, two and three examinations per year.

**Table 1:** Time period (years) required to detect various rates of MD change with 80% power in visual fields with low, moderate and high degrees of variability with one (a), two (b) and three (c) examination per year

(a) 1 examination/year Progression rate (dB/year)	Variability		
	Low	Moderate	High
−0.25	13	19	30
−0.5	9	13	19
−1.0	6	9	13
−2.0	5	6	9

(b) 2 examinations/year Progression rate (dB/year)	Variability		
	Low	Moderate	High
−0.25	6.5	8.5	15
−0.5	4.5	6.5	8.5
−1.0	3	4.5	6.5
−2.0	2.5	3	4.5

(c) 3 examinations/year Progression rate (dB/year)	Variability		
	Low	Moderate	High
−0.25	4.3	6.3	10
−0.5	3	4.3	6.3
−1.0	2	3	4.3
−2.0	1.7	2	3

**Table 2:** Rates of visual field change corresponding to total change in mean deviation (MD) over 2, 3 and 5 years (a) and the number of visual fields per year required to detect the corresponding change with 80% power (b)

(a) Total MD change (dB)	Progression rate (dB/year)		
	2 years	3 years	5 years
−1.0	−0.5	−0.3	−0.2
−2.0	−1.0	−0.7	−0.4
−4.0	−2.0	−1.3	−0.8

(b) Total MD change (dB)	Annual examinations		
	2 years	3 years	5 years
−1.0	7	6	4
−2.0	5	4	3
−4.0	3	3	2

## OTHER REQUIREMENTS FOR MEASURING VISUAL FIELD PROGRESSION

### Examination quality

Examination quality is important. Automatic trust in the reliability indices produced by the software should be avoided. Besides false-positive rates, other indices (fixation losses and specifically false negative rates) may not accurately reflect examination quality. Consideration should be given to other indicators of examination quality, such as the perimetrist’s notes (if available), patient attention, artefacts such as lens rim defects, poor centration, incorrect refractive correction, etc, before including or discarding examinations to evaluate progression. High-quality visual field examinations often depend on perimetrists being well informed, trained and motivated. Learning effects can sometimes be difficult to account for, as they can be negligible, or persist over several examinations. Generally speaking, unless there are obvious explanations, significant improvements in the initial period of follow-up can be attributed to learning.

### Examination strategy

Visual field change should only be determined with examination strategies that estimate thresholds, such as Swedish Interactive Thresholding Algorithm (SITA) and full threshold bracketing strategies. The same strategy (eg, always SITA Standard) and test pattern (eg, always 30–2 or always 24–2, and not a mixture of, for example, 30–2 and 10–2) is always much better for determining visual field change.

### Progression software

Visual field examinations generate a considerable amount of data. Software programs that perform objective and automated analyses are available and should be used.^27^^–^30 All reliable examinations should be used in the analyses to measure progression accurately. Decision-making based on examination of grey-scale plots should be avoided.

## CONCEPTS THAT ERRONEOUSLY HINDER THE USE OF SAP TO MEASURE PROGRESSION

While stressing the importance of measuring visual field progression, the evidence supporting some commonly held views should be re-examined.

### Is SAP sensitive enough?

It is often quoted that up to 50% of retinal ganglion cells (RGCs) are lost before onset of visual field damage.^31^ More recent studies with SAP reported lesser degrees of RGC loss.^32^ ^33^ Many methodological issues regarding scaling of visual field measurements^34^ and localised losses of RGCs (which make up a smaller proportion of overall RGC loss but are more readily detectable by SAP^32^) make SAP measurements relevant. The sensitivity of imaging devices to identify early glaucomatous visual field loss at a specificity of 90–95% is around 70%.^35^^–^37 The ability of clinicians to identify structural glaucomatous damage is similar to that of the imaging devices.^38^ ^39^ This means that automated or clinical evaluation of structural damage fails to identify around 30% of eyes with established early visual field loss.

### Is detection of early visual field loss important?

Visual field loss is strongly associated with quality-of-life measures.^1^^–^6 Recent evidence shows that motor vehicle accidents are significantly related to the level of SAP loss,^40^^–^42 in spite of patients being well within the legal visual requirements to hold a driving licence and having only early field damage in the *worse* eye.^40^

### Are visual field examinations difficult to perform reliably?

It is often thought that visual field examinations are difficult to perform. This attitude can potentially transfer to ophthalmology trainees, staff and patients. Inappropriate or inaccurate instructions to patients can lead to anxiety and unreliable results. For example, perimetrists and patients should be made aware that during a typical examination, around 50% of the stimuli are not expected to be seen. Appropriate and standardised patient instructions produce reliable and usable results in the vast majority of patients.^43^ ^44^

### Can visual fields be performed with cataract and macular degeneration?

Cataract and macular degeneration are often co-morbidities of glaucoma. The effect of mild to moderate cataract on the visual field can be small or even negligible,^45^ ^46^ though more advanced cataract can have a larger impact.^47^ ^48^ While cataract can confound the glaucomatous visual field, software to largely remove its influence is available.^27^ ^49^ Macular degeneration will, to a certain extent, change the appearance of central glaucomatous visual field defects and their progression. Central visual fields should always be corroborated with fundus examination. Tools to aid patient fixation in automated perimetry are available and should be used.

### Can small amounts of visual field progression be detected?

The amount of visual field change that can be detected depends on the variability and number of available examinations, as described above. Both theoretical and empirical evidence indicates that small amounts of clinically significant change can be detected with an adequate number of examinations and sufficient follow-up time.^50^ ^51^

### Are other perimetric techniques more sensitive than SAP?

Recent evidence supporting the notion that perimetric techniques such as SWAP and FDT perimetry are clearly more sensitive at detecting field progression is weak.^52^ ^53^ While there is active research in this area, it is recommended that for routine practice, other perimetric techniques should be considered adjunct and not be performed at the expense of SAP.

### Are structural tests more sensitive markers of the disease?

Recent research shows weak concordance between visual field and structural measures of progression.^26^ ^54^ In routine practice, given a certain number of tests, it is more effective to perform the same test more frequently than a variety different tests less frequently. Evidence supporting earlier detection of progression with optic disc and RNFL measurements is difficult to obtain.^26^ Examination of the optic disc and RNFL is of unquestionable importance providing additional and complementary information about disease progression. However, if resources are limited, modern structural tests should not be substituted for SAP examinations if the frequency of the latter falls below recommended levels.

### Are measurements of structure more reliable because they are objective?

Because obtaining optic disc or RNFL images does not require a subjective response from the patient, they may be erroneously considered “real” and without measurement error. However, these measurements can vary because of many patient- and instrument-related factors. Furthermore, the frequency of imaging required to identify progression is comparable with that for visual fields.^55^ ^56^ Similar to analyses of visual fields, variability can confound the interpretation of change.^57^ ^58^

## RECOMMENDATIONS

On the basis of the discussion above, we make the following recommendations.

### Perform sufficient examinations to detect change

The progression status of the patient is unknown unless there is enough information. Table 2(a) shows the rate of MD change corresponding to total change in MD over 2, 3 and 5 years in visual fields with moderate variability. The frequency of examinations per year required to detect the corresponding amounts of MD change is shown in table 2(b). 

### Six visual field examinations should be performed in the first 2 years

This rules out the presence of rapid progression (−2 dB/year or worse) and establishes a good set of baseline data.

### Measure the rate of visual field progression

Estimating the rate of progression is invaluable for guiding therapeutic decisions and estimating the likelihood of visual impairment during the patient’s lifetime. In most cases, establishing visual field progression requires several years.

### Use the same threshold test

Any analysis of progression can only be performed if the same threshold algorithm and test pattern is used.

### Pay attention to examination quality

Examinations of poor quality will likely lead to an erroneous assessment of progression. There should not be automatic reliance on the reliability indices. Unless there are other reasons (such as obvious learning effects, high false-positive errors, rim artefacts) examinations should not be removed from the analyses.

## CONCLUDING REMARKS

Detection of visual field progression requires understanding of variability, the magnitude of change that is considered clinically significant and the number of visual fields required to detect this change with adequate statistical power. In addition to rates of change, other approaches such as event-based analyses are also available and useful in certain clinical situations. Clinical decisions on patient management require more than a formulaic approach based on visual field progression because risk factors such as baseline damage, age, and IOP may have different relative weights in driving these decisions. For this reason, these recommendations are not a protocol, but a practical guide and template for the examination frequency to detect different rates and/or magnitudes of change, as well as other considerations for the appropriate use of perimetry for detecting glaucomatous progression.
